# Biomechanical validation of additively manufactured artificial femoral bones

**DOI:** 10.1186/s42490-022-00063-1

**Published:** 2022-08-04

**Authors:** F. Metzner, C. Neupetsch, A. Carabello, M. Pietsch, T. Wendler, W.-G. Drossel

**Affiliations:** 1 ZESBO Centre for Research on Musculoskeletal Systems, Leipzig University, Semmelweisstraße 14, 04103 Leipzig, Germany; 2 Department of Orthopaedics, Trauma and Plastic Surgery, Leipzig University, Leipzig, Germany; 3 Fraunhofer Institute for Machine Tools and Forming Technology, Dresden, Germany; 4 Professorship of Adaptronics and Lightweight Design, Chemnitz Universtiy of Technology, Chemnitz, Germany

**Keywords:** Artificial bone, Additive manufacturing, Femoral, Bone model, 3D-printing, Femur, Biomechanics, Hip, 3d-printing

## Abstract

Replicating the mechanical behavior of human bones, especially cancellous bone tissue, is challenging. Typically, conventional bone models primarily consist of polyurethane foam surrounded by a solid shell. Although nearly isotropic foam components have mechanical properties similar to cancellous bone, they do not represent the anisotropy and inhomogeneity of bone architecture. To consider the architecture of bone, models were developed whose core was additively manufactured based on CT data. This core was subsequently coated with glass fiber composite. Specimens consisting of a gyroid-structure were fabricated using fused filament fabrication (FFF) techniques from different materials and various filler levels. Subsequent compression tests showed good accordance between the mechanical behavior of the printed specimens and human bone. The unidirectional fiberglass composite showed higher strength and stiffness than human cortical bone in 3-point bending tests, with comparable material behaviors being observed. During biomechanical investigation of the entire assembly, femoral prosthetic stems were inserted into both artificial and human bones under controlled conditions, while recording occurring forces and strains. All of the artificial prototypes, made of different materials, showed analogous behavior to human bone. In conclusion, it was shown that low-cost FFF technique can be used to generate valid bone models and selectively modify their properties by changing the infill.

**Supplementary Information:**

The online version contains supplementary material available at 10.1186/s42490-022-00063-1.

## Background

Bone is a composite material consisting of a solid outer shell (cortical bone) and a foam-like interior (cancellous bone) filled with bone marrow. According to Wolff’s law, bone tissue continuously adapts to current loading situations, resulting in a highly optimized structure characterized by a high degree of anisotropy and inhomogeneity [1–4]. Orientation and density of trabeculae significantly determine the mechanical properties of bones [5].

Conventionally manufactured bone models like the biomechanical models from Sawbones (Sawbones, Pacific Research Lab, Vashon Island, Washington, USA) consist of two main components. Polyurethane foam is used as cancellous bone substitute, which can be manufactured in various degrees of hardness. These foams generally have equivalent stiffness and strength as cancellous bone and are inexpensive to manufacture. However, in contrast to bone, foams exhibit an almost isotropic and homogeneous material behavior [6], which means that they only reproduce the characteristic trabecular structure to a limited extent. To substitute cortical bone, a harder foam or fiber-reinforced plastics are used, depending on the intended application [7–9].

Additive manufacturing (AM) processes are a possible alternative enabling novel design approaches depending on the process being used. Fused-filament-fabrication (FFF), in which an object is built up in layers, is one of the most common AM processes due to relatively low material and equipment costs, as well as a wide range of materials [10]. Therefore, it is selected for the present study.

Additively manufactured bone models are mostly used in clinical applications for visualization and orientation of complex bone defects or tumors [11–13]. Those models validly reproduce the geometry of the anatomy to be imaged [14–16]. However, so far there are only limited quantitative data on the mechanical behavior of printed bone models. Previous studies evaluated the haptic sensation when working with the bones [17, 18] or used very specific, non-standardized test methods to evaluate bone models [19, 20].

The following research questions result from the limitations described above: Can FFF be used to generate structures having the same or similar mechanical behavior as human bone (i) and do the developed bone models show comparable interaction with implants (ii)?

Hence, the goal is to evaluate biomechanical bone models generated by AM using standardized material tests. Moreover, the models’ performance will be validated based on a clinically relevant scenario.

## Method

To reproduce the anisotropy and inhomogeneity of a bone structure, bone models consisting of two components were generated: An additively manufactured core reproducing the mechanical properties of cancellous bone was combined with a fiber-reinforced composite within the shaft region of the femoral bone. A bottom-up approach was used for validation. First, the individual model components (cancellous and cortical parts) were subjected to uniaxial material tests. After assembling the model components, biomechanical tests were carried out both on the artificial bones and on human bones.

### Cancellous bone

**Fig. 1:**
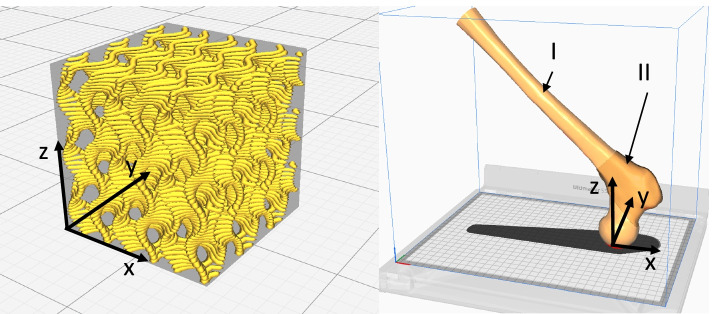
Preview of a cube with the gyroid structure (left) and the model core (right) shown in the Ultimaker Cura software. The printing direction corresponds to the z-axis of the coordinate system. The extruder moves in x and y direction. The femoral bone model is oriented such, that the z-axis is aligned with the main oriantation of cancellous bone within the femoral neck. Two different zones with varying infill are highlyghted with roman numbers

The additively manufactured core of the model consists of multiple zones (s. Fig. 1, right), exhibiting various amounts of infill, mimicking the heterogeneity of cancellous bone. The trabecular structure of cancellous bone is approximated using a gyroid-structure (s. Fig. 1, left) provided by the pre-processing software Ultimaker Cura (V 4.8). All parts are fabricated with an Ultimaker S5 (Ultimaker B.V., Utrecht, Netherlands) using 0.4 mm nozzles and a layer thickness of 0.15 mm with recommended printing parameters for each material.

10 cubes each were made of polylactic acid (PLA) with 10 mm edge length and densities of 30, 40 and 50% without outer walls for investigating the anisotropy of the spongy component (s. Fig. 1). The cubes were then divided into two groups and tested in spatial directions z and x/y using an uniaxial compressive test. Due to the layer-by-layer structure of the FFF process, the generated gyroid structure is assumed to be orthotropic. This means that the material behavior in x and y direction is identical. In later stages of the study, the process-induced anisotropy was utilized in the following way. The artificial bones were aligned within the 3D printer in a manner that the main direction of the trabeculae corresponded to the z-direction.

Mechanical validation of the filling structure was carried out next. Five cylinders (Ø 8x16 mm) each with relative densities between 20 and 50% without outer walls (s. Fig. 1) were manufactured additively from different materials and investigated using uniaxial compression tests as before. The longitudinal axis of the cylinders pointed in the z-direction (see Fig. 1). The materials include acrylonitrile butadiene styrene (ABS), PLA, polycarbonate (PC) and polymethyl methacrylate (PMMA). Material specification and manufacturing parameters are listed in Table 1.

**Table 1:** Summary of the applied materials and their specific settings used for additive manufacturing

	PLA	PC	ABS	PMMA
**Extruder Temperature (°C)**	205	280	250	260
**Build Platform Temperature (°C)**	60	110	85	100
**Fan Speed (%)**	100	0	2	20
**Material Specification**	Polylite PLA	Polylite PC	ABS Premium	3DIAKON™
**Manufacturer**	Polymaker, Suzhou, China	Polymaker, Suzhou, China	Verbatim GmbH, Eschborn, Germany	Mitsubishi Chemical Corporation, Tokyo, Japan

**Fig. 2:**
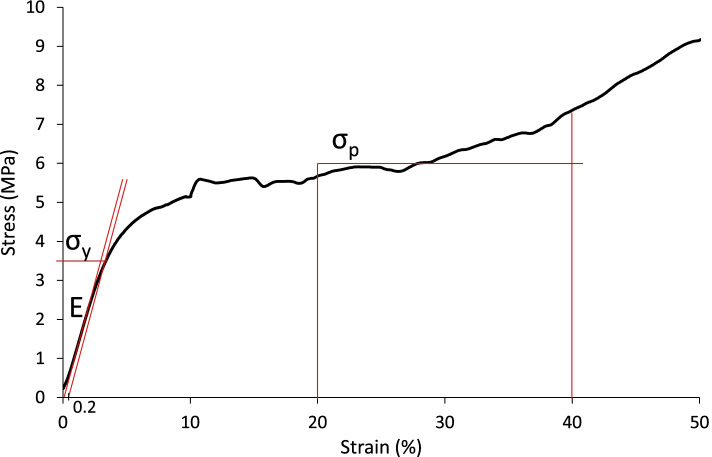
Stress-strain curve of a specimen with 30% infill made of PC. No initial stress maximum could be detected, so the yield stress σ_y_ was determined by offsetting the modulus by 0.2% strain

All compressive tests were performed according to Metzner et al. [21] and the results were compared with the mechanical properties determined for cancellous bone specimens (Ø 8x16 mm) from the proximal femur. Specimen were grouped according to the WHO classification for osteoporosis, harvested along the trabecular orientation of the femoral neck and tested in uniaxial compression [21]. Compressive modulus E was calculated from the maximum slope in the linear-elastic region of the stress-strain curve. The maximum stress σ_max_ was defined as the first maximum in the stress-strain curve. If there occurred no initial stress maximum yield stress σ_y_ was determined at 0.2% offset of the modulus (s. Fig. 2). Plateau stress σ_p_ was defined by the average of all stress values between 20 and 40% strain. Tests stopped at 50% strain.

Cancellous bone heterogeneity was realized throughout the bone model by applying two zones of different densities. The proximal zone was generated with the infill that most closely approximated the strength of the human bones, which is quantified in the results. A minimal infill of 5% was applied in the diaphysis zone as an internal support for reliable manufacturing of the outer walls. Only the proximal two third of the femur was manufactured, since the entire bone did not fit into the build volume of the printer. Furthermore, the condyles were not required for biomechanical testing. All generated core elements had a wall thickness of 1.0 mm.

**Fig. 3:**
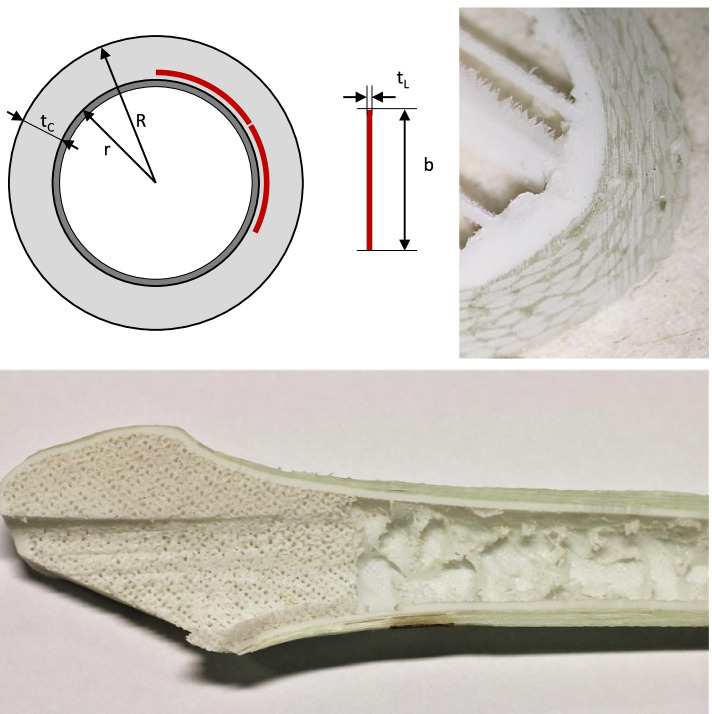
Schematic cross-section with inner radius r and outer radius R of the composite. The lined-up laminate strips with thickness t_L_ and width b are shown as red bars (upper left). Upper right shows a transverse section of the prototype. The bottom picture shows a frontal section running through the PC model after biomechanical testing. Wall thickness tapers from the diaphysis towards the proximal parts. Additionally, two zones of different infill densities (shaft and proximal femur) are displayed

The geometry of the cancellous bone substitutes was based on CT data of a human femur (length: 494 mm; head diameter: 50 mm; CCD angle: 121 °) which was segmented using Materialise Mimics software (Materialise, Leuven, Belgium). Cortical thickness was measured at several points along the length of the diaphysis and a mean diaphysis thickness of 7 mm was determined. This was applied globally to simplify design and fabrication. The outer contour of the 3D-model was thinned in the area of the diaphysis using the erode-function. Afterwards, he removed area was replaced by applying the glass fibre composite to restore the original bone contour (s. Fig. 3). A separate volume was created for the area of the proximal femur reaching from the femoral head to the trochanter minor. After that, all Models were imported into Ultimaker Cura and merged to an assembly. This is possible as all volumes are extracted from the same set of CT images and are therefore referenced to the same coordinate system. Zones with varying infill density were implemented using a software tool for defining object properties for overlapping volumes in the preprocessing software.

### Cortical Bone

The composite is made of unidirectional glass fibre strips (R&G Faserverbundwerkstoffe GmbH, Waldenbuch, Germany) and epoxy resin. The computation of the composite’s stiffness was based on the young’s modulus by using the rule of mixtures [22]. Based on the anatomy of the bony template, the cross-sectioned area of the femoral shaft to be laminated is considered a hollow cylinder with a thickness of 7 mm. The required fiber volume content was calculated by rearranging the equation for the linear mixing rule (1) by using the stiffness characteristics of the glass fiber strips (E_F_ = 73 GPa), resin (E_M_ = 3.2 GPa) and the target value of the composite E_C_ of 18 GPa (s. Table 3) for femoral cortical bone (s. Table 3)..

1 $${E}_C={E}_F\bullet \varphi +{E}_M\bullet \left(1-\varphi \right)=18\ GPa$$

Transforming the equation yields the required relative fiber volume content φ_rel_:

2 $${\varphi}_{rel}=\frac{E_C-{E}_M}{E_F-{E}_M}=0.21$$

Given the wall thickness t_W_ = 7 mm of the femoral diaphysis, the relative fiber thickness t_F_ is

3 $${t}_F={t}_W\bullet {\varphi}_{rel}=1.47\ mm$$

The fiber volume content φ =35% [22] which is achievable by hand lay-up leads to the required composite thickness t_C_

4 $${t}_C=\frac{t_F}{\varphi }=4.2\ mm$$

According to the manufacturer, the thickness of a single laminate layer t_L_ is 0.55 mm for hand lay-up. Therefore, seven layers are required to achieve a composite thickness of 4.2 mm. The composite was applied longitudinally to the shaft in individual 30 mm wide laminate strips, with the fiber direction corresponding to the longitudinal axis of the femoral diaphysis. The core has an average perimeter of 58 mm which corresponds to an idealized circle with a radius of r = 9.2 mm. Adding the required laminate thickness t_C_ results in a circular ring with the outer diameter R = 13.4 mm (s. Fig. 3).

The cumulative cross-section of n strips wrapped around the core must be equal to the cross-section of the circular ring. The number of required strips (n) is thus calculated as follows

5 $$n=\frac{\pi \left({R}^2-{r}^2\right)}{t_L\bullet b}=18.1.$$

Therefore, 18 strips were applied to the core. Protruding fibers were processed with sandpaper after curing and overhanging laminate strips at the distal end were trimmed.

The cortical model component was validated using 3-point-bending tests. A prototype laminate core with the required outer geometry but without a specific filler structure was generated using the FFF process and reinforced with the fiber composite. Block-shaped specimens (2x10x40 mm) of the prototype and fresh-frozen human femora of three donors were extracted using a band saw (Exakt 310, EXAKT GmbH Norderstedt, Germany) from the mid diaphyseal shaft according to [23, 24]. Flexural strength, flexural modulus, and flexural strain were determined according to DIN ISO 178.

### Biomechanical testing

A femoral arthroplasty stem was implanted in the prototypes as well as in three human femoral bones (75.3 ± 4.0 years) from male body donors in order to validate the artificial models. The bones where freshly frozen after harvesting and stored at −80 °C. Artificial models were named according to the core material being used and the human bones (HB) by sequential numbers.

**Fig. 4:**
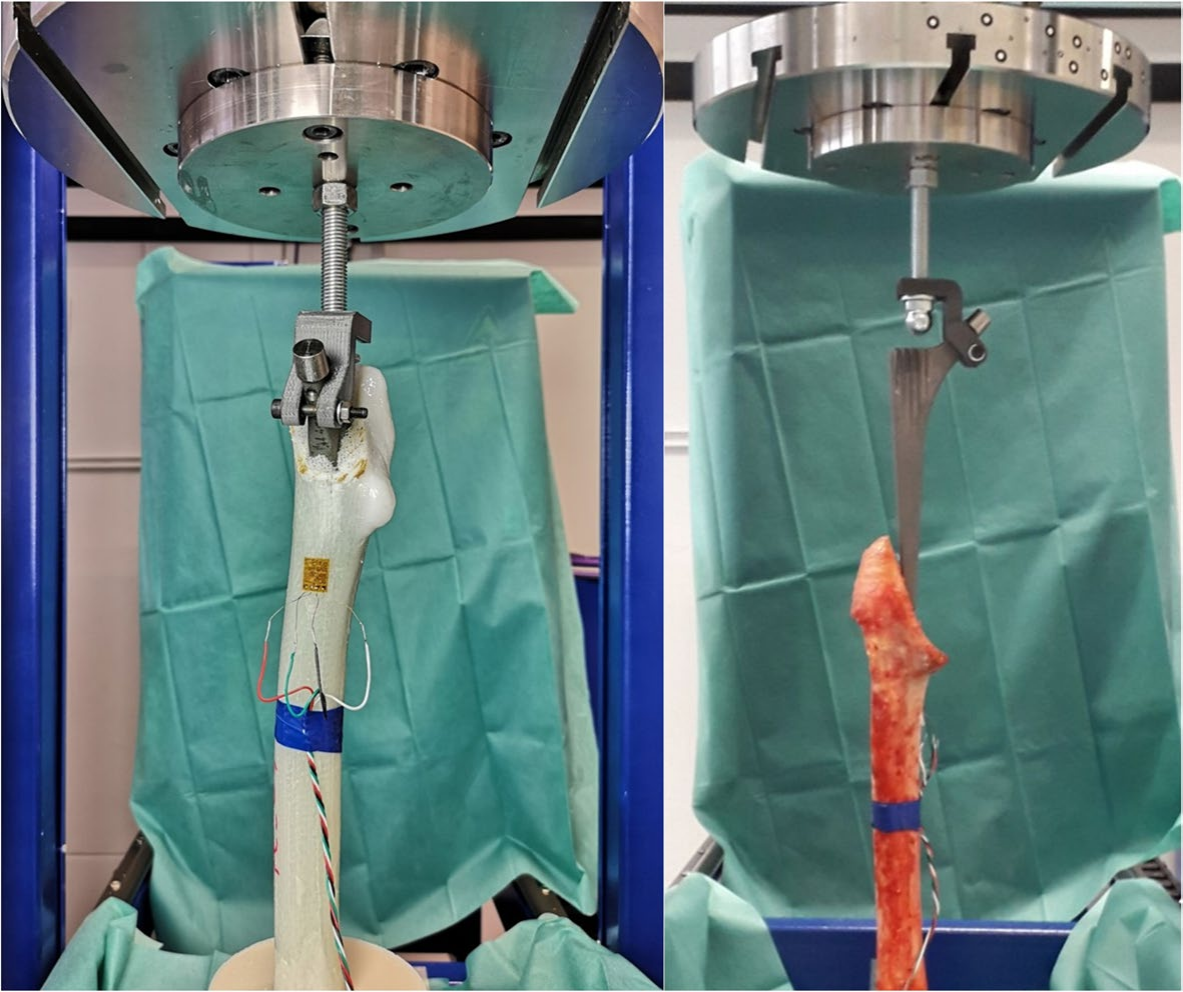
A sensored artificial bone (left) and a human bone (right) in the testing machine. The load isapplied via a threaded rod. An additively manufactured device holds the implant in place during insertion and is removed before testing

Implantation started with removing the femoral head using a band saw. The cutting line is oriented alongside the linea intertrochanterica and runs perpendicular to the frontal plane through the femur as shown in Fig. 4 (right). Afterwards, the bones were fixed in anatomical position with a casting resin (Rencast FC52a/B aluminum hydroxide DT0821, Huntsman International LLC, The Woodlands, TX, USA) and a strain gauge (SGT-4/1000-FB13, Omega Engineering, Deckenpfronn, Germany) was applied medially to the shaft (s. Fig. 4) [25]. Next, the bone was prepared with surgical rasps and then the stem was pressed into the bone. During surgery, this is performed by using a hammer and impactor tool. Here, this procedure was carried out in a testing machine (F_max_ = 10 kN, DYNA-MESS Prüfsysteme GmbH, Aachen/Stolberg, Germany) in order to reproduce the insertion of the stem as accurately as possible as well as to determine the point of failure.

Prior to testing, the embedded and sensored bone was vertically aligned in the testing machine and the prosthesis stem was inserted into the medullary canal without any load (see Fig. 2). The crosshead of the testing machine incrementally moved upwards with intervals of 5 mm at a speed of 5 mm/s until failure. The force was applied vertically via a stainless steel screw with a ball head attached at the designated point on the implant. Surface strain was recorded by the applied strain gauge sensors. In addition, force and displacement data were recorded by the testing machine. Testing was terminated when a maximum force of 9 kN occurred or the specimen failed visibly. Maximum strains and forces, as well as subsidence were determined as comparison parameters.

## Results

### Cancellous bone

The cubes made of PLA showed higher values for the mechanical properties (E, σ_y_, σ_p_) in z-direction than in x/y-direction, as shown in Table 2 in terms of mean (X̅) and standard deviation (S̅). Similarly, all mechanical properties increase with an increase in density. The dependency of materials on direction is called the degree of anisotropy (DA) and is considered separately for all mechanical parameters. The compressive modulus E has a DA of 2 to 3 for all investigated specimens. DA ranges from 1.2 to 1.8 for the strengths σ_y_ and σ_p_.

**Table 2:** Detailed results of the compression tests with the cube-shaped PLA specimen. Mean value (X̅) and standard deviation (S̅)

				E (MPa)		σ_**y**_ (MPa)		σ_**p**_ (MPa)	
	Infill	Test direction	***n***	***X̅***	***S̅***	***X̅***	***S̅***	***X̅***	***S̅***
Printed cubes	30%	**X/Y**	5	61.3	17.0	2.3	0.9	3.5	0.7
		**Z**	5	179.3	28.1	4.3	0.7	6.4	0.7
	40%	**X/Y**	5	187.8	24.6	5.7	0.5	7.9	0.7
		**Z**	5	392.2	82.7	6.9	0.9	10.6	1.4
	50%	**X/Y**	5	385.2	66.4	8.2	1.0	12.1	1.3
		**Z**	5	1084.1	237.4	11.2	3.3	17.7	1.8

Selecting the infill for the cores was based on the compressive tests with the manufactured cylindrical specimens (s. Fig. 4) by comparing them to human bone with corresponding specimen size from the proximal femur [21]. Not all manufactured specimen showed a local stress maximum (σ_max_) like cancellous bone does. Therefore, failure stress σ_max_ of the bone specimens was directly compared with σ_y_ of the plastic specimen.

**Fig. 5:**
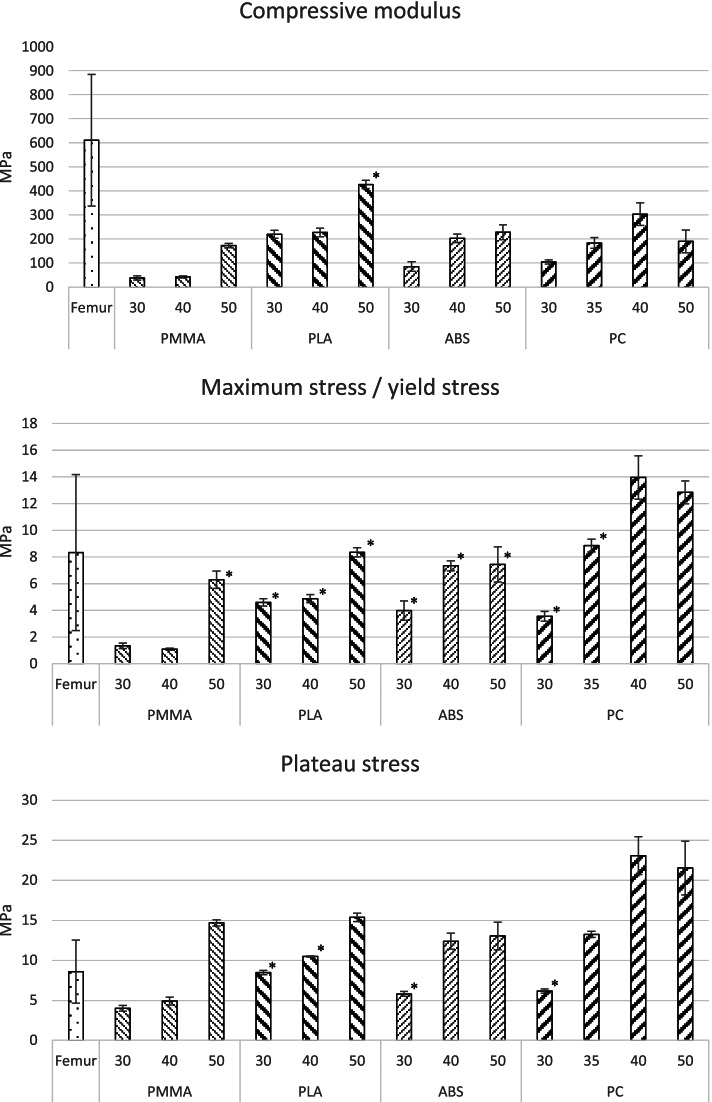
Mechanical properties of the additively manufactured cylinders and the human reference values [21]. The marked values correspond to the condition that the limits of the 95% confidence intervals of the plastic specimen are within the standard deviation of the human specimen

Strength and stiffness of the specimens increase generally with increasing infill, exept the specimen made with PC and 50% infill. It showed lower values for all mechanical properties compared to the ones made with 40% infill (s. Fig. 5). An additional set of PC samples were manufactured since corresponding groups with 30 and 40% infill had great differencesin their mechanical properties. Except for PLA sample with 50% infill as well as PC sample with 40% infill, the moduli of all plastic specimens were lower compared to the bony model of 612 ± 270 MPa.

Equivalence tests were carried out to check whether the mechanical properties of the plastic specimens were within the range of the human specimens. For all characteristic values, it was checked whether the limits of the 95% confidence intervals of the plastic specimens lie within the standard deviation of the human specimens. Equivalent values are marked in Fig. 5. The averaged σ_y_ of each group is within the standard deviation of the human comparison values, except for the PMMA specimens with 30 and 40% infill. The values of σ_p_ are about 1.5 to 2 times greater than their corresponding σ_y_ (or σ_max_). An exception were the ABS specimens with 30% infill. In contrast, the plateau stress of the human comparison group is slightly higher than the maximum stress.

For the materials ABS, PLA and PMMA, 40% infill and for PC 35% infill was chosen as their infill level at the proximal femur for generating the model cores. Finally, based on the knowledge gained regarding the anisotropy of the generated gyroid-structure, the models were aligned in the build volume with the femoral neck oriented in the z-direction and the femoral head resting on the build platform.

### Cortical bone

The 3-point bending tests showed the laminate component to have 1.8 times higher modulus, 3.25 times higher flexural strength, and 1.3 times higher flexural strain than bone. Detailed results are listed in Table 3.

**Table 3:** Results of the 3-point bending tests showing mean (X̅) and standard deviation (S̅) of flexural modulus (E_b_) flexural strength (σ_b_) and flexural strain (ε_b_) of human bone specimen and specimen gathered from the composite

		E_**b**_ / (MPa)		σ_**b**_ / (MPa)		ε_**b**_ / (%)	
	n	X̅	***S̅***	X̅	***S̅***	X̅	***S̅***
**Bone**	12	18,389.3	3130.4	182.1	45.5	1.7	0.3
**Composite**	6	33,112.5	6607.3	592.8	131.5	2.3	0.4

### Biomechanical testing

It appeared that the generated cancellous bone component of the PLA model began to melt locally while being machined during preparation of the medullary canal. During insertion of the femoral stem, it was found that human bone specimens resisted to the highest forces of up to 9 kN. Problems with the strain gauge sensor were encountered when measuring specimen HB3 (s. Table 4), so strain data is not available for it. Furthermore, the test of HB1 was stopped after a subsidence of 25 mm was reached due to suspected fracture. All further tests were terminated when fractures occurred visibly. Table 4 lists the maximum force, strain and subsidence of each specimen. Highest force and strain were measured for the specimen HB2.

**Table 4:** Maximum values of subsidence and maximum insertion force (F_max_), as well as surface strain (ε_max_) for the respective specimens during the biomechanical tests (^a^ testing was aborted due to suspected fracture)

		Subsidence (mm)	ε_**max**_ (%)	F_**max**_ (kN)
**Human Femur**	HB1	35	0.019	3.9
	HB2	30	0.164	8.5
	HB3	25	–	4.2^a^
**Composite Bones**	PC	35	0.131	3.8
	PLA	35	0.144	4.7
	ABS	40	0.052	4.7
	PMMA	25	–	5.0

**Fig. 6:**
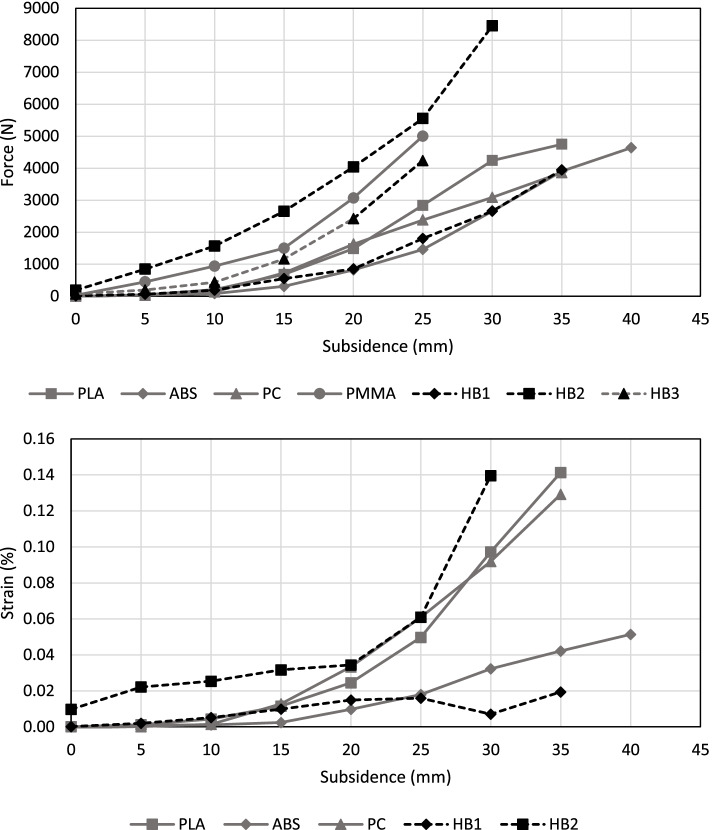
Insertion force and surface strain plotted against the subsidence of the implant into the bone. Each increment resembles the corresponding local maximum value

The graphs in Fig. 6 show all local maximum values for each increment. Force and strain increase continuously and nonlinearly with the subsidence, except specimen HB1 Here, the surface strain decreases from 0.016 to 0.007% at 30 mm subsidence and then rises to a value of 0.02%. The unflattened test data (s. supplementary material) show that the strain drops to 0.004% at a subsidence of 25 mm and further decreases to −0.009% at 30 mm before reaching a maximum value of 0.019%. The graphs in the appendix show these moments as force and strain peaks in the curves, and they become more pronounced as the test duration progresses. Generally, there are significant variations in both force and strain at the respective subsidence. However, almost all measured values are within the range of the bone specimens.

## Discussion

### Cancellous bone

FFF technology was chosen due to its wide range of materials and low-cost end devices. Internal structures in the FFF process do not have to be manually designed in contrast to stereolithography, selective laser melting or laser sintering. Instead, different structures as well as the amount of infill can be configured during software-assisted pre-processing.

DA for the manufactured cube-shaped specimen ranges from roughly 1 to 3. Lowest values were observed for the specimen with 40% infill. Augat et al. [26] investigated the anisotropy of human cancellous bone by testing cubes extracted from different bone regions in several spatial directions. Tested specimen from the proximal femur showed a DA of 0.8 and 6.2 and a mean value of 2.2. Comparable results were provided by Goulet et al. [27], where DAranged from 1.1 to 2.5. Thus, the anisotropy of the artificial models corresponds to human bone very well.

All cylindrical specimens have a lower compressive modulus compared to human bone. This is to be expected, due to the large discrepancy between the moduli of the used plastics and that of bone tissue of about 18 GPa or more [24, 28, 29].

When testing human cancellous bone in compression, the stress-strain curve shows a maximum stress at initial failure, which immediately decreases to about 2/3 of the maximum. At higher strains, bone is characterized by a periodic progression of increasing and decreasing stresses around a roughly constant plateau stress, until the specimen becomes compacted, resulting in a rapid stress increase [30]. Typically, the plateau stress is less than or equal to the maximum stress depending on anatomical site and bone density [21, 31]. With increasing relative density compaction occurs at lower strains and thus may be noticeable by a sharp increase in plateau stress. In this sense, the discrepancy between failure stress σ_y_ and σ_p_ is included for the selection of suitable infills.

The absence of maximum stress at initial failure of the synthetic specimens may be due to the viscoelastic material behavior of the used thermoplastic materials. The material behavior could additionally be caused by the generated gyroid structure being a pure shell structure. In contrast, the bone architecture is a cellular structure, containing both platelike and rodlike struts, depending on the anatomical position [32]. The absence of rods in the gyroid structure could have an effect on the mechanical behavior. This could be one cause of major difference in mechanical behavior of human bone and gyroid structures made of plastic.

Silva et al. [33] investigated the influence of the gyroid structure on the mechanical stability of additively manufactured components in different loading directions (tension, compression, bending, impact strength). Among other things, the influence of the infill density on the mechanical load-bearing capacity of cylindrical specimens with and without an outer wall was analyzed. For PLA specimens with 50% infill σ_max_/ σ_y_ is about 10 MPa and E about 800 MPa [33]. In the present study, cube-shaped and cylindrical specimens made of PLA were tested. Compared with the data of Silva et al., the cube-shaped specimens in the z-direction show a higher average σ_max_ (11.8 ± 3.3 MPa) and E (1084.1 ± 237.4 MPa). In contrast, the cylindrical specimens have both lower σ_y_ (8.4 ± 0.3 MPa) and E (427.9 ± 17.6 MPa) than the data of Silva et al.

Applying the gyroid structure as a cancellous bone substitute holds great potential. In addition, there is the possibility of transferring the modelling approach to other body regions since the manufacturing settings can be precisely adjusted and the mechanical properties of the object show good reproducibility. Most of the tested specimen have equivalent σ_y_ compared to human bone (s. Fig. 5). For example, mechanical properties of PMMA compression specimens with 30 and 40% infill show very good agreement with cancellous bone specimens from the lumbar spine [34]. In addition, cancellous bone in vertebral bodies exhibits nearly orthotropic material behavior, as do the generated specimens.

The selection of suitable filling grades is based, on the one hand, on the ratio of σ_p_ to σ_y_ and, on the other hand, on the highest possible modulus. With the exception of PC, applying 40% infill was chosen. For the PC 35% infill was used.

### Biomechanical testing

Removing the femoral head with a saw and preparing the medullary canal provided a subjective evaluation of the human bones. The specimens made of PC, PMMA and ABS could be instrumented better than the model made of PLA due to their higher thermal resistance.

Considering the local force maxima over subsidence (s. Fig. 5), all artificial models are within the range of the human specimens. All specimens show a nonlinearly increasing force peaks with increasing implant subsidence. Specimen HB2 exhibits the steepest increase in the force curve and reaches the highest measured insertion force of 8.5 kN without visible fractures. It can be assumed that this specimen would have reached even higher forces but the test was stopped at this value to protect the load cell.

An explanation for the low values of the ABS specimen may be the low bonding forces between the deposited layers of the additively manufactured core. As a result, the components exhibit significantly lower mechanical properties in the x/y-direction than in the z-direction. Consequently, the subsidence of the prosthesis into the bone has increased and the occurring loads are comparably low over time. Both force and displacement of the ABS model show very good agreement with the specimen HB1 during testing.

The actual forces occuring during the insertion of a femoral stem, as well as the forces leading to calcar fractures are poorly investigated. Some studies report proximal femoral fractures were caused by pressing in an oversized stem using a testing machine, but do not state the required forces [35, 36]. Sakai et al. [37] reported hammering forces of 9.25 kN in their in vitro biomechanical study using artificial bones. However, by using a very rigid experimental setup, without damping properties of human tissue, these forces seem to be too high.

To the authors’ knowledge, the only comparable data were published by Carls et al. [38]. They also pressed femoral stems into human femora utilizing a testing machine and recorded the force progression and subsidence until failure. Their results, similar to those in the present study, show a wide range of failure forces between 1.9 kN and 9.3 kN or subsidences between 2.0 mm and 19.1 mm.

An equivalent increase in surface strain was generally recorded on the inner surface of the femoral stem during insertion. The measured strains are up to 0.14%. An exception is specimen HB1, where inverted strains were observed. The inversion of strain into a local compression could be explained by a lateral fracture of the bone. A crack in the bone causes the femur to bend up radially in cross-section, resulting in local compressive loads on the bone surface. As long as the bone is intact, increasing insertion force leads to an increase of the local strain.

### Limitations

Evaluating the anisotropy of the gyroid structure was only performed on specimen made of PLA. It is to be expected that the DA varies among different materials. Based on the collected data, it is assumed that all materials have higher mechanical properties in the z-direction than transversely and that DA varies only in magnitude.

The inhomogeneity of cancellous bone was simulated by two zones of different infill density. In real bone, this homogeneity is much more complex. Nevertheless, the use of AM, especially FFF, allows a much higher variability of mechanical properties compared to PU foams. Another limitation is the small number of specimen used in the biomechanical testing. Although the data do not allow statistically relevant conclusions, the insertion tests still provide information that the model approach is working.

Another issue regarding the manuctured cubic and cylindrical specimen is the ratio of pore size and specimen size. Another issue regarding the manufactured cubic and cylindrical specimen is the ratio of pore size and specimen size. The mechanical response of porous structures loaded in uniaxial compression are dependend on the ration of specimen diameter and pore size. Tekog̃lu et al. [39] showed, that mechanical properties decrease with decreasing ratio of specimen diameter and pore size. Since the mechanical properties of many additively manufactured specimens were lower than the human comparative data, it can be assumed that a wide range of materials and filling grades might be applicable for mimicking human cancellous bone. A reliable parameterization regarding human cancellous bone properties requires more targeted investigations due to the above-mentioned systematic errors.

Manufacturing components using the hand lay-up technique is simple and inexpensive, but it also involves a high probability of defects such as cavities (Fig. 3, top right). The stiffness-reducing influence of such defects cannot be ruled out in the conducted experiments. It can also be assumed that the transversal mechanical stability of the glass fiber laminate does not exceed the stiffness of the polymer matrix (3.2 GPa). Human femoral bone, on the other hand, has a stiffness of 11.5 Gpa [40]. This could be an explanation for the lower insertion forces of the artificial bones. In this study, the glass fibers were intentionally oriented only longitudinally to provoke tearing (periprosthetic fracture) of the stem during implantation, as this is a common complication [41]. An optimization of the glass fiber laminate would be necessary for improving the artificial model in future.

## Conclusion

Additively manufactured bone models are used in clinical research and patient care more frequently. Currently bone models are mainly used to visualize complex bone defects. Patient-specific models generated by AM deepen the understanding of bone defects or diseases for both medical staff and patients compared to virtual reconstructions and analyses of three-dimensional imaging such as CT and MRI [11–13]. The performed experiments provide an overview of the mechanical properties of the gyroid structure generated by the FFF method for several materials. With the presented approach human cancellous bone can be accurately imitated, particularly by creating areas of varying infills. Furthermore, the reinforcement of generated bone models using fiber-plastic composites allows tubular bones to be realistically replicated.

The findings highlight a new dimension for the application of AM technology in clinical practice. A valid representation of the mechanical properties of artificial bone models can be used, for example, to compare different treatment approaches in advance to complex surgeries.

## Supplementary Information

**Additional file 1.**

## Data Availability

All data generated or analysed during this study are included in this published article and its supplementary information files.
